# *In vivo* / *in vitro* Correlation of Pharmacokinetics of Gentamicin, Vancomycin, Teicoplanin and Doripenem in a Bovine Blood Hemodialysis Model

**DOI:** 10.3389/fphar.2021.702455

**Published:** 2021-06-24

**Authors:** M G Vossen, S Pferschy, C Milacek, M Haidinger, Mario Karolyi, Zoltan Vass, Heinz Burgmann, Alexandra Maier-Salamon, S G Wicha, W Jäger, M Zeitlinger, T Stimpfl, T Wittek, F Thalhammer

**Affiliations:** ^1^ Clinical Division of Infectious Diseases and Tropical Medicine, Department of Medicine I, Medical University of Vienna, Vienna, Austria; ^2^ Department of Internal and Emergency Medicine, Bürgerspital Solothurn, Solothurn, Switzerland; ^3^ Department for Infectious Diseases, Sozialmedizinisches Zentrum Sued Kaiser-Franz-Josef-Spital, Wien, Austria; ^4^ Department of Pharmaceutical Chemistry, Division of Clinical Pharmacy and Diagnostics, University of Vienna, Vienna, Austria; ^5^ Department of Clinical Pharmacy, Institute of Pharmacy, University of Hamburg, Hamburg, Germany; ^6^ Department of Clinical Pharmacology, Medical University of Vienna, Vienna, Austria; ^7^ Department of Laboratory Medicine, Medical University of Vienna, Vienna, Austria; ^8^ University Clinic for Ruminants, University of Veterinary Medicine Vienna, Vienna, Austria

**Keywords:** bovine blood, pharmacokinetics, chronic hemodialysis, clearance, antimicrobial agent

## Abstract

**Background:** Elimination of a drug during renal replacement therapy is not only dependent on flow rates, molecular size and protein binding, but is often influenced by difficult to predict drug membrane interactions. *In vitro* models allow for extensive profiling of drug clearance using a wide array of hemofilters and flow rates. We present a bovine blood based *in vitro* pharmacokinetic model for intermittent renal replacement therapy.

**Methods:** Four different drugs were analyzed: gentamicin, doripenem, vancomicin and teicoplanin. The investigated drug was added to a bovine blood reservoir connected to a hemodialysis circuit. In total seven hemofilter models were analyzed using commonly employed flow rates. Pre-filter, post-filter and dialysate samples were drawn, plasmaseparated and analyzed using turbidimetric assays or HPLC. Protein binding of doripenem and vancomycin was measured in bovine plasma and compared to previously published values for human plasma.

**Results:** Clearance values were heavily impacted by choice of membrane material and surface as well as by dialysis parameters such as blood flow rate. Gentamicin clearance ranged from a minimum of 90.12 ml/min in a Baxter CAHP-170 diacetate hemofilter up to a maximum of 187.90 ml/min in a Fresenius medical company Fx80 polysulfone model (blood flow rate 400 ml/min, dialysate flow rate 800 ml/min). Clearance of Gentamicin vs Vancomicin over the F80s hemofilter model using the same flow rates was 137.62 mL vs 103.25 ml/min. Doripenem clearance with the Fx80 was 141.25 ml/min.

**Conclusion:** Clearance values corresponded very well to previously published data from clinical pharmacokinetic trials. In conjunction with in silico pharmacometric models. This model will allow precise dosing recommendations without the need of large scale clinical trials.

## Introduction

The kidneys play a vital role in the detoxification of the human body as well as in blood pressure regulation and endocrinologic balance. Detoxification is performed through a complex combination of filtration, osmosis and active transport. Simplified, blood passes a semi permeable “membrane” (formed by the podocytes in the glomerulus) and, following the created pressure gradient, parts of the plasma are forced through the membrane while the corpuscular part and the larger proteins are held back. The basic principle behind medical hemodialysation is diffusion of soluble molecules through a semipermeable membrane. Other than in the human glomerulum, the detoxification is achieved by passive diffusion following an osmosis gradient rather than filtration along a pressure gradient (Pea et al., 2007). Substances which have accumulated in the patients’ blood diffuse through the membrane into a saline solution, the dialysate fluid. Since there is no active transport, elimination is influenced by the composition of the dialysis fluid, the pore size and surface area of the membrane as well as flow rate of blood and dialysis solution. Standard high-flux dialysis membranes remove molecules with a size up to 50 kDa (Pea et al., 2007). The relationship between molecular weight and removal is inverse proportional - as the molecular weight increases the removal decreases. Low-molecular weight solutes diffuse easily through the pores of any available membrane.

Drug therapy in patients receiving hemodialysis or hemodiafiltration always possesses the risk of under- or overdosing the administered drug due to unknown elimination through the membrane. Elimination of a given drug during hemodialysis is not easily predictable. As a consequence, most prescribing information supply little guidance on how to prescribe antimicrobials in hemodialysis patients. Even highly protein bound drugs such as ceftriaxone, which should not be eliminated during hemodialysis, may interact with the filter itself and thus be eliminated via the formation of a “secondary protein membrane” which forms by adhesion of proteins to polysulfone (Patel et al., 1984; Keller et al., 1987; Soriano, 1992; Meyer et al., 2003). It is important to note that different membranes have very different adhesion profiles for proteins as well as drugs, thus affecting the drug plasma level directly and indirectly (Francke et al., 1979; Nikolaidis and Tourkantonis, 1985; Davies et al., 1988; Traunmüller et al., 2002; Bouman et al., 2006). To avoid under- as well as over dosage, the obvious solution would be repeated drug level monitoring for the drug in use, which unfortunately is only available for a very limited number of agents, and is usually only feasible in larger hospitals (Kroh et al., 1996; Sowinski et al., 2001).

As a consequence pharmacokinetic trials are needed for each drug, and would preferably be performed at least for the most commonly employed membrane types with a wide variety of blood and dialysate flow rates. Considering that each trial should at least include 10 patients to achieve sufficient statistical power, a high number of patients would thus potentially be exposed to either insufficient or toxic drug concentrations. As a consequence a precise model allowing prediction of *in vivo* clearance would be beneficial.

The optimal model substance would be human whole blood. Considering that at least 2 L of whole blood would be needed, the models’ use would be limited by the necessary expenses. Non-corpuscular solutions such as albumin in PBS however lack important pharmacokinetic components: pure saline lacks both potential drug-albumin and drug-erythrocyte interaction. We thus aimed at the development of a model featuring the full spectrum of serum proteins with a similar protein binding of drugs as human plasma and including erythrocytes. Bovine blood has been used as model substance for renal replacement therapies before, however this is to our knowledge the first time it has been used as model substance for intermittent renal replacement therapy (Chaijamorn et al., 2017; Schneditz and Daugirdas, 2020; Andrews et al., 2021). It is uniquely qualified for simulation of intermittent renal replacement therapy as hematocrit values in bovine blood correspond to those found in patients undergoing intermittent renal replacement therapy (Pstras et al., 2019).

The model described in this manuscript aims at the *in vitro* simulation of drug pharmacokinetics during hemodialysis. The nature of the model allows to fully characterize elimination of any given drug through a number of different membranes and flow rates, thus reducing patient numbers needed in clinical trials. To allow for external validation of the model, we reproduced the dialysis conditions of earlier trials within the model. Thus each membrane-drug combination was run with previously published flow rates as well as with a standardized flow rate to allow comparability.

## Materials and Methods

Hemodialysis was simulated using a Fresenius 4008H dialysis machine. After priming of the extracorporeal circuit including the dialysis membrane with 0.9% saline solution, a reservoir bag containing 2 L of 37°C warm heparinized bovine blood (provided in cooperation with the University of Veterinary Medicine Vienna after seeking authorization by the institutional and governmental ethics committee, (ZI.04/04/972,013, GZ: 68.205/0240-WF/V/3b/2016) was connected to the circuit. The blood was kept at 37°C throughout the experiment. It was mixed with 2,000 IU of low molecular weight heparin directly during the blood draw as well as during the experiment itself with a rate of 500 IU per hour. After circuit connection, the investigated drug was added to the reservoir in order to mimic plasma levels previously described in trials (Lanese et al., 1989; Thalhammer et al., 1997; Amin et al., 1999). The targeted concentrations were as follows: gentamicin 10 mg/L, vancomycin 20 mg/L, teicoplanin 25 mg/L, doripenem 40 mg/L. After shaking of the reservoir, the extracorporeal circuit was primed with blood, discarding the sodium chloride solution left in the tubing. After priming the system with blood, it was allowed to run for 30 s to allow equilibration of blood hematocrit throughout the extracorporeal circuit before the baseline sample was drawn. Due to the reduced volume compared to *in vivo* and the comparably large hemofilter membrane area, a considerably faster drug concentration decline was expected. Thus, pre filter, post filter and ultrafiltrate samples were collected at the end of minutes 1, 5, 10, 15, 20, 30, 40 and 60 of the run. All samples were plasma separated by centrifugation at 2000 ×vg for 7 min and the supernatant stored at −80°C for analysis.

An actual blood flow rate of 300 ml/min and a dialysate flow rate of 500 ml/min were kept as standard flow rates. Depending on previous literature, single drugs were run at different flow rates to allow external validation of the model. Ultrafiltration (corresponding to negative fluid balance/water removal during hemodialysis) was kept at 0 ml/min. Table 1 shows the flowrates and membranes for each drug. Hemodialysation and hemofiltration experiments were performed with Baxter Gambro CAHP 170 diacetate devices, Nipro Sureflux 17UX and 21UX triacetate membranes as well as Fresenius Hemoflow F80s and FX 80 polysulphone membranes. Vancomycin and teicoplanin were additionally measured with Fresenius F60s polysulphone membranes as previous publications have described the pharmacokinetics using this membrane (Lanese et al., 1989; Thalhammer et al., 1997).

**TABLE 1:** Clearance values measured for gentamicin, vancomycin, teicoplanin and doripenem *in vitro*.

	Gentamicin								Vancomycin					Teicoplanin		Doripenem	
Membrane	SF21E	SF17UX	SF21UX	F80s	F80s	Fx80	Fx80	CAHP-170	F60s	F80s	F80s	F80s	Fx80	F60s	Fx80	SF21UX	Fx80
BFR (ml/min)	400	400	400	300	400	300	400	400	250	250	300	400	300	250	250	300	300
DFR (ml/min)	800	800	800	500	800	500	800	800	500	500	500	800	500	500	500	500	500
Cl (ml/min)	131.52	160.75	178.25	122.37	137.62	142.13	187.90	90.12	71.20	83.35	93.61	103.25	91.11	25.50	43.49	155.76	141.25
SD	16.04	26.09	46.13	9.95	12.15	6.03	13.24	31.55	4.91	19.81	31.11	18.34	7.14	17.7	23.06	18.65	22.92
N	12	12	17	12	12	12	12	16	16	8	12	12	12	16	12	12	12

BFR, blood flow rate in ml/min; DFR, dialysate flow rate in ml/min; Cl, *in vitro* clearance rate in ml/min; corrected by hematocrit; SD, standard deviation.

Plasma protein samples from two cows were stored in advance and the same two samples used for all bovine plasma protein binding experiments. Pooled Human fresh frozen plasma was bought from Europlasma (Lot# A010013900137) and aliquoted. All human plasma protein binding experiments were performed using these aliquots. Analysis of plasma protein binding for Vancomycin was performed as ultrafiltration of the plasma water by centrifugation of a 1 ml aliquot in a Millipore Centrefree device (Lot# R4DA37544, Merck Millipore Ltd. Ireland) at 2000 × g for 10 min in a fixed angle rotor and measurement of plasma water concentration of the drug in relation to its serum concentration.

Plasma protein binding of doripenem was analyzed by using Centrisart I Ultrafiltration Devices (Sartorius Stedim Biotech S.A., Aubagne, France) at 2000 × g for 20 min at room temperature. The recovered ultrafiltrate was analyzed by high performance liquid chromatography to determine the concentration of free (unbound) drug in the plasma. Samples that did not undergo ultrafiltration were assayed to determine total (bound and unbound) drug concentration.

Serum and ultrafiltrate concentration of the investigated drug was measured by high performance liquid chromatography at the Department of Pharmaceutical Sciences at the University of Vienna, Austria (doripenem, described in (Vossen et al., 2018) ) or by CE certified assay based on kinetic interaction of microparticles in a solution using a Cobas c501 device at the Department of Laboratory Medicine of the General Hospital of Vienna, Austria (Cobas GENT2 Online TDM Gentamicin, Cobas VANC3 Online TDM Vancomycin Gen.3 (Roche Diagnostics GmbH), QMS Teicoplanin (Microgenetics Corporation, Thermo Fisher Scientific Inc.).

The measured drug plasma concentrations were analyzed to calculate the area under the curve, hemofilter clearance and the drug saturation coefficient. As removal within the hemofilter is expected to be too fast for equilibration of drugs out of the erythrocyte compartment and drug concentrations were measured in the plasma, all clearance values were calculated with a hematocrit corrected formula, in which Hc is the blood hematocrit in percent divided by 100, BFR the actual blood flow rate, C_pre_ the drug concentration measured before the blood enters the filter and C_post_ the drug concentration measured after the filter. Additionally the formula allows for correction of blood concentration by ultrafiltration (UFR).

$$\left(1-Hc\right)\times BFR\times \frac{\frac{C_{pre}-\left(C_{post}\left(BFR-UFR\right)\right)}{BFR}}{C_{post}}$$

Hematocrit corrected and not corrected clearance, total “body” clearance, volume of distribution, area under curve, elimination coefficient and half-life were evaluated for their correspondence to previously published *in vivo* values. Whenever previous publications declared the method of clearance calculation, the same formula was used. As a foreseeable consequence of the reduced blood volume compared to patients and the missing deeper compartments only hematocrit corrected and not corrected clearance measured pre-filter/post-filter showed any approximation to historical data, while data such as plasma concentration at or after the end of hemodialysis did not correspond to *in vivo* data. As such, only clearance parameters were analyzed further on. An empirical fitting of the *in vitro* PK curves and available *in vivo* PK curves was performed to gain an insight into the time relation between *in vitro* and *in vivo*.

More recent historic data on intradialytic gentamicin clearance only exists for the Baxter CAHP-210 membrane (Sowinski et al., 2008) and the Fresenius F80s (Amin et al., 1999) which are both not available anymore. Baxter however was kind enough to supply us with several CAHP-170 and F80s models. To extrapolate assumed clearance of a CAHP-210, we compared clearance gains between a Nipro Sureflux 17UX and a Sureflux 21UX membrane. The factor between the 1.7 m^2^ and the 2.1 m^2^ membrane was 1.2. Assuming the same factor applies to the structurally related CAHP models, we tried to extrapolate an assumed clearance of the CAPH-210 from our CAPH-170 clearance data.

## Results

**FIGURE 1:**
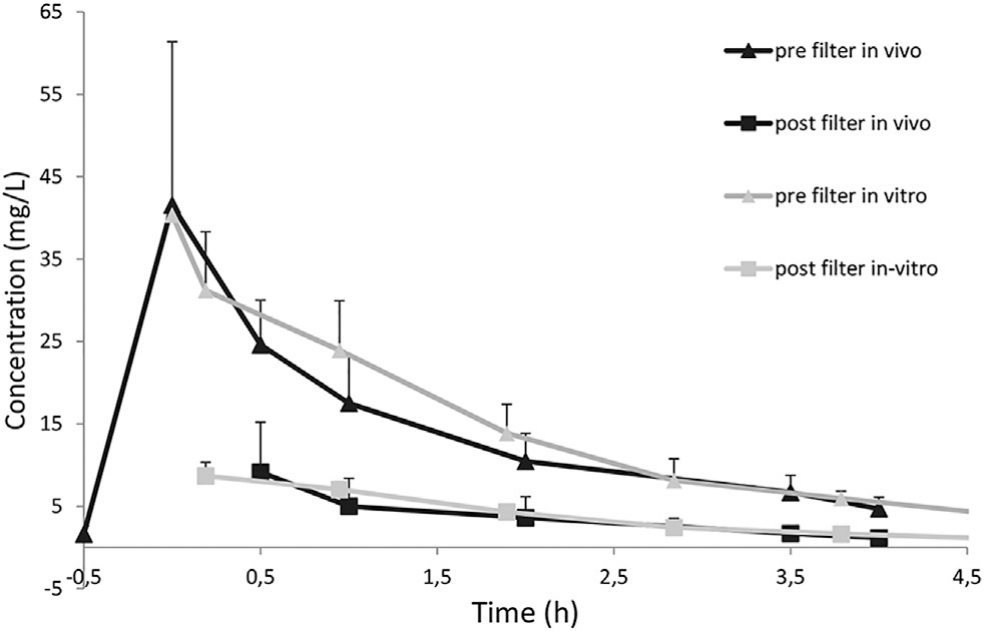
Doripenem drug concentrations during *in vivo* and *in vitro* hemodialysis. *In vitro* timepoints were multiplied by a factor of 11.36 which is the ratio between Vd *in vivo* and *in vitro*. Whiskers indicate standard deviation. *In vivo* data has been published in 2018 [16].

**FIGURE 2:**
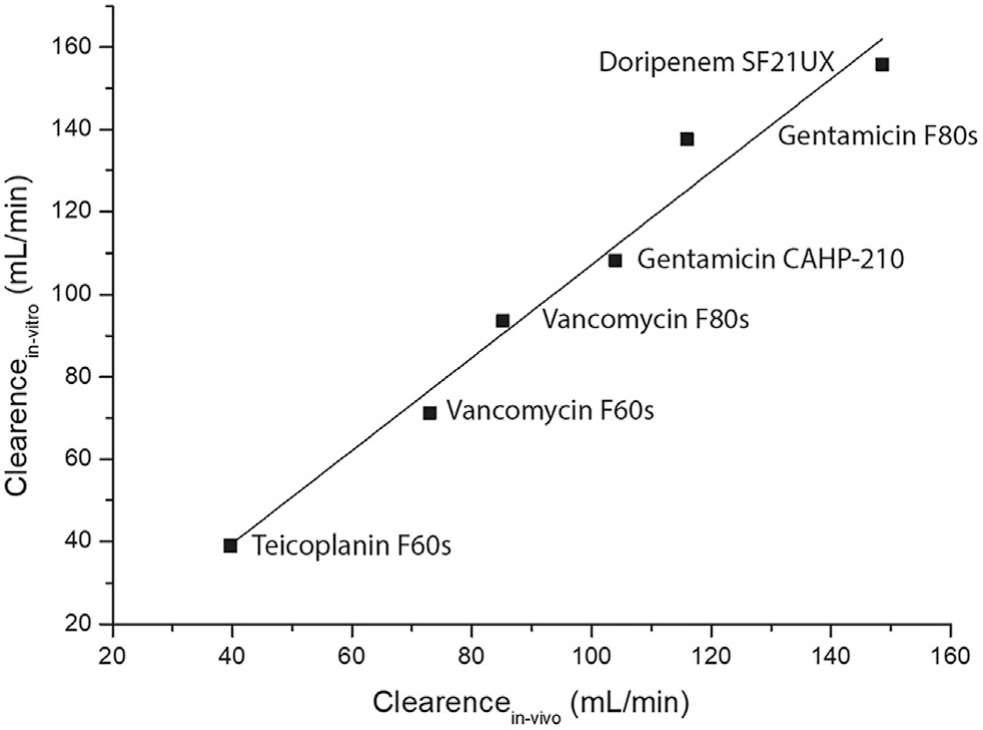
Best-fit straight-line correlation graph between previously published *in vivo* hemodialysis clearance rates and the corresponding results from the *in vitro* experiments. Number of Points 6, Degrees of Freedom 4, Residual Sum of Squares 2,43,25,364, Pearson’s r, 0,98667, Adj. R-Square 0,96689

After the initial setup of the test circuit, hemodialysis of gentamicin, teicoplanin and vancomycin was performed to validate the test setup and evaluate optimal timepoints for analysis of serum concentration. As expected serum concentrations of all drugs dropped considerably faster than *in vivo* as a consequence of the smaller absolute drug quantity in the system. However when fitted with a time multiplication factor of 11.36, calculated as the ratio between Vd *in vitro* and Vd *in vivo* the doripenem pk curves aligned quite well (Figure 1). Clearance rates for gentamicin, vancomycin, teicoplanin and doripenem showed a good correlation with previously published data (Table 2). This is also apparent in a correlation diagram with best-fit straight line (Figure 2). For teicoplanin the non-hematocrit corrected clearance had to be used, as clearance rates in the comparator trial were not corrected for hematocrit. An additionally performed simulation of teicoplanin clearance with a Sureflux SF 21E triacetate dialyzer showed very low clearance values of 17.6 ml/min.

**TABLE 2:** Comparison of *in vitro* and *in vivo* clearance values. Clearance values in this table were calculated as described in each referenced original publication.

	Gentamicin		Vancomycin		Teicoplanin^a^	Doripenem
Membrane	CAHP-210	F80s	F60s	F80s	F60s	SF21UX
Cl_*in*_ _*vitro*_ [ml/min]	108.34^b^	137.62	71.20	93.61	38.95	155.76
SD [ml/min]	24.32^b^	12.15	4.91	31.11	26.73	18.65
Cl_*in*_ _*vivo*_ [ml/min]	104.03 Sowinski et al. (2008)	116 Amin et al. (1999)	73.0 Lanese et al. (1989)	85.2 Lanese et al. (1989)	39.70 Thalhammer et al. (1997)	148.61 Vossen et al. (2018)
SD [ml/min]	12.44	9	5.0	7.0	24.50	8.41

a Teicoplanin *in vitro* clearance: non hematocrit corrected clearance, teicoplanin *in vivo* clearance: non-hematocrit corrected clearance, recalculated using original data from the manuscript by F.Thalhammer (Thalhammer et al., 1997).

b Data extrapolated from CAHP-170 clearance values (see Table 1). Cl_*in*_ _*vitro*_, *in vitro* clearance rate, corrected by hematocrit if not otherwise specified; Cl_*in*_ _*vivo*_, clearance as described in referenced publication; SD, standard deviation.

**FIGURE 3:**
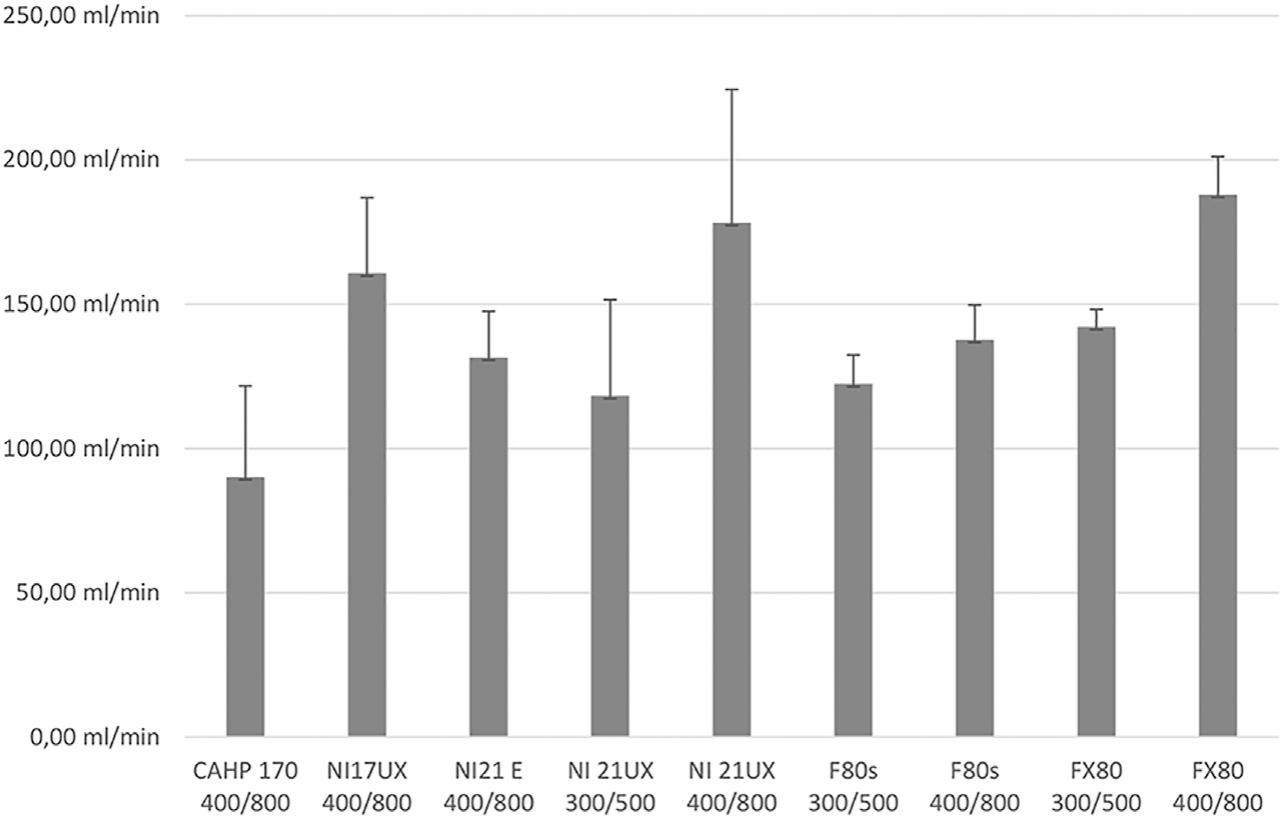
Different Gentamicin clearances found in different membranes and flow rates. Membranes: CAHP 170: Baxter high performance cellulose diacetate membrane, 1.7 m^2^ surace area; SF17UX: Nipro Surflux-17UX high performance cellulose triacetate membrane, 1.7 m^2^ surface area; SF21UX: Nipro sureflux-21UX high performance cellulose triacetate membrane, 2.1 m^2^ surface area; SF21E: Nipro Sureflux-21E balanced medium-flux cellulose triaceate membrane, 2.1 m^2^ surface area; F80s: Fresenius medical care F80s, high flux polysulphone membrane, 1.8 m^2^ surface area; FX80: Fresenius medical care FX 80classix high flux polysulphone membrane, 1.8 m^2^ surface area. Flow rates are given below the membrane identifier: first number represents bloodflow in ml/min, second number dialysate flow in mL/min. Standard deviation is identified by whiskers.

For gentamicin an extensive clearance profile with different membranes and flow rates was performed (Figure 3). We could show an increased clearance with increased flow rates as well as illustrate the effect of modern membrane technologies. This may be demonstrated by the comparison of the CAHP-170 diacetate membrane clearance compared to the much increased clearance of the Nipro 17UX triacetate membrane or the increase of clearance between the FMC F80s to the advanced model Fx80.

Plasma protein binding values of vancomycin and doripenem showed very good agreement between historic human data and measured protein binding in bovine plasma (Table 3). Earlier publications have reported a slightly higher bound fraction of vancomycin of 54.8% (±3.0) (PMC, 1972). Protein binding for teicoplanin could not be performed due to the very high (<99%) plasma protein binding of this drug and the resulting low teicoplanin concentration in the ultrafiltrate (Dykhuizen et al., 1995). Gentamicin does only exhibit minimal and very variable plasma protein binding (Gordon et al., 1972; Kirby, 1977; Bailey and Briggs, 2004). The measured clearance values can be found in Table 1.

**TABLE 3:** Comparison of plasma protein binding of vancomycin and doripenem between previously published values and binding rates found in bovine blood.

	Vancomycin	Doripenem
Human	41.5% Butterfield et al. (2011)	8.0% HORI et al. (2006)
Bovine	43.6%	9.7%

## Discussion

Current dosing recommendations in patients undergoing renal replacement therapy are usually based on small pharmacokinetic trials in patients. As a result of the ever increasing complexity of such trials, dosing recommendations once made are seldom updated with more recent membranes, possibly leading to underexposure to the administered agent as a result of increased clearance. This is easily explained with the data presented in Figure 3, where more recent membrane designs show an increased gentamicin clearance compared to older designs.

Our data shows that the elimination of most drugs is in large parts dependent on the membrane area. However, large evolutionary steps in membrane materials, such as the step from diacetate membranes such as the Baxter CAHP family to intermediate flow triacetate membranes as the Nipro Sureflux-E and the next step to high flow triacetate membranes such as the Nipro Sureflux-UX (SFUX) family have caused considerable impact on clearance rates, as is shown in Figure 3. Similar to Decker *et al.* we found overall considerably increased clearance values compared to previous publications (Decker et al., 2012). However, due to the larger membrane area of the dialyzers used in our trial the clearance rates presented in this manuscript are even higher, further supporting administration of high doses of aminoglycosides pre dialysis and complete removal of the drug during hemodialysis, thus reducing toxicity (Decker et al., 2012; Veinstein et al., 2013; Vossen and Thalhammer, 2013). Only for certain drugs with a strong and specific binding to membrane materials – as is the case in teicoplanin–elimination is dominated by membrane material. This behavior seems to be connected to the large molecule size as well as high protein binding. Plasma protein binding alone does not seem to be a reliable predictor of drug membrane interaction or clearance rates. In contrast the higher clearance rates of vancomycin compared to teicoplanin corresponded well with the increased protein binding of teicoplanin.

The *in vitro* dialysis setup described above, allows to predict drug-membrane interaction and clearance rates on a much more granular level than *in vivo* trials in patients. The good match between *in vitro* and *in vivo* clearance rates will allow to perform *in vitro* estimation of drug elimination and thus help to predict necessary drug dosing in dialysis patients. Together with an *in silico* model of dialysis clearance, this data will in the future allow computer based dosing recommendations for the individual patient (Broeker et al., 2020).

Few limitations shall be acknowledged: A drawback of our *in vitro* model is the necessity to use freshly drawn bovine blood for each experiment. Running multiple drugs in one experiment is possible, however interaction between substances potentially influencing filtration or adhesion to the membrane is not easily predictable in all cases. As a result reduction of the used amount of bovine blood is a challenge. Moreover blood clots which may sometimes develop during the blood drawing procedure within the needle or tubing will, if not removed from the system, often plug parts of the filter thus invalidating the clearance result of this specific experiment. Additionally, only hemofilter clearance can be described by the model, and as mentioned initially drug levels drop much more rapidly than *in vivo* allowing only the measurement of clearance rates with high certainty. Lastly, the model will not take post-hemodialytic rebound effects into account.

In conclusion, the good correlation between *in vivo* and *in vitro* clearance data enables *in silico* renal replacement modality dependent dose recommendations without the need to expose large patient populations to experimental treatment in clinical trials.

Additionally, this model enables us to provide independent comparisons between dialysis efficacy and drug clearance of different membranes and dialysis modes.

*In vitro* simulation of intermittent dialysis is a valuable addition to the pharmacokinetic armamentarium, improving dose calculation for *in vivo* pharmacokinetic trials and allowing valuable insight into drug-membrane interactions of both old and newly developed drugs.

## Data Availability

The raw data supporting the conclusions of this article will be made available by the authors, without undue reservation.

## Abbreviations

AUC, area under the curve; BFR, blood flow rate; Cl, clearance; DFR, dialysate flow rate; GFR, glomelular filtration rate; HD, hemodialysis; HDF, hemodiafiltration; HPLC, high performance liquid chromatography; iRRT, intermittent renal replacement therapy; Q_B_, Blood flow; Q_D_, Dialysate flow.
